# Fusion of computational and experimental provenance in RO-Crate

**DOI:** 10.1515/jib-2025-0050

**Published:** 2026-07-06

**Authors:** Caroline Ott, Kevin Schneider, Heinrich Lukas Weil, Florian Wetzels, David Zimmer, Christoph Garth, Timo Mühlhaus

**Affiliations:** Department of Biology, Computational Systems Biology, RPTU Kaiserslautern-Landau, D-67663 Kaiserslautern, Germany, https://csbiology.github.io/; Department of Computer Science, Visualization & HCI, RPTU Kaiserslautern-Landau, D-67663 Kaiserslautern, Germany, https://vis.cs.rptu.de/

**Keywords:** FAIR, FDO, RO-Crate, workflows, research data management

## Abstract

Modern research projects typically involve several measurement techniques and computational analyses, resulting in complex, multimodal research data objects. While the FAIR Principles provide a framework for making data Findable, Accessible, Interoperable, and Reusable, researchers must still apply community standards for each data type, where available. This challenge is particularly pronounced in computational analyses and workflows, where high methodological diversity makes it difficult to create meaningful FAIR Digital Objects that also capture experimental provenance. Such objects must describe the full research process by interlinking experimental and computational steps in a unified, queryable provenance model. The ISA framework captures laboratory protocols and processes, while computational analyses are described using workflow-centric models such as Workflow (Run) RO-Crate, which separate workflow definitions from execution traces. Despite conceptual similarities, these approaches remain largely disconnected. This paper presents the ARC Workflow Run RO-Crate profile, which aligns ISA-based experimental provenance with computational workflow and execution metadata in a single, standards-compliant container. It enables shared semantics and makes experimental assays and workflow runs interoperable within a coherent provenance graph interpretable by both ISA- and workflow-aware tools. At the same time, the profile harmonizes experimental and computational provenance and remains compatible with existing (Workflow) RO-Crate and ISA tooling ecosystems.

## List of abbreviations

FDO: *FAIR Digital Object*
FAIR: *Findable, Accessible, Interoperable, and Reusable*
ISA: *Investigation Study Assay*
WROC: *Workflow RO-Crate*
WRROC: *Workflow Run RO-Crate*
ARC: *Annotated Research Context*
ARC WRROC: *Annotated Research Context Workflow Run RO-Crate*
RO-Crate: *Research Object Crate*
OPMW: *Open Provenance Model for Workflows*
CWL: *Common Workflow Language*
SOP: *Standard Operating Procedure*
WMS: *Workflow Management System*
SemVer: *Semantic Versioning*

## 1 Introduction

The *Findable, Accessible, Interoperable, and Reusable* (FAIR) Principles call for research outputs to be packaged as machine-actionable *FAIR Digital Object*s (FDOs) that expose their content, context, and provenance in a structured and interoperable manner [1]. In practice, constructing such FDOs remains challenging for projects that combine experimental measurements with computational analyses. While both domains rely on rich provenance to support reuse, traceability, and automation, they are typically described using distinct target-specific metadata models and tooling ecosystems. This results in a fragmented representation of a single research process with the individual parts only interpretable by the respective, intended endpoints.

*Research Object Crate*s (RO-Crates) are a lightweight, JSON-LD-based approach for packaging methods, data, outputs and the outcomes of projects with rich, customizable metadata [2]. It enables tailoring the generic RO-Crate packaging format to specific domains, use cases, or community standards using RO-Crate profiles. These profiles constrain that structure: they specify required entity types, properties, and serialization formats ensuring consistency and enabling tools to rely on predictable patterns [3].

For computational analyses, workflow-centric approaches have emerged as a dominant solution. The *Workflow RO-Crate* (WROC) profile enables consistent description of such workflow definitions, including their inputs, outputs, and software environments, and supports registry-based discovery through platforms such as WorkflowHub [2], 4]. Its *Workflow Run RO-Crate* (WRROC) profile extension complements this by capturing retrospective provenance of workflow executions using CreateAction-based traces, thereby distinguishing between prospective workflow specifications and their concrete executions [5]. Together, these profiles support the creation of executable and reproducible computational FDOs.

In experimental research, provenance is commonly captured using the *Investigation Study Assay* (ISA) framework, which distinguishes between investigation (project context and administrative metadata), studies (units of research) and assays (analytical measurements). Studies and Assays record parametrized laboratory processes, connecting source materials and results in a formally linked process graph [6]. ISA emphasizes the distinction between planned activities (protocol) and performed processes (in the processSequence), enabling fine-grained traceability across experimental steps. This model has proven effective for representing wet-lab workflows and was recently mapped by us into the RO-Crate ecosystem through the ISA RO-Crate profile using the BioSchemas types LabProtocol and LabProcess introduced for this purpose [7], [8], [9].

Despite their shared goals and conceptual similarities, ISA-based experimental metadata and workflow-based computational provenance remain largely disconnected. Computational workflows are typically documented in terms of executability and runtime behavior, while experimental workflows emphasize contextual annotations. As a consequence, FDOs that aim to represent complete research lifecycles often resort to parallel or loosely linked provenance descriptions, limiting integrated querying, cross-domain reasoning, and reuse.

This paper addresses this gap by presenting a unified RO-Crate profile that aligns experimental and computational workflow descriptions within a single provenance model. Building on the WROC, WRROC, and the ISA RO-Crate profiles, we introduce the *Annotated Research Context Workflow Run RO-Crate* (ARC WRROC) Profile, which establishes shared process semantics across domains by systematically multi-typing workflow specifications and executions. Computational workflows are simultaneously described as ComputationalWorkflow and LabProtocol, while workflow runs are represented as both CreateAction and LabProcess. This alignment allows experimental assays and computational workflow runs to be treated as first-class, interoperable entities within one coherent metadata graph.

The resulting profile enables the construction of a single, standards-compliant FDO that captures prospective and retrospective provenance across experimental and computational steps without sacrificing compatibility with existing tools. Workflow-aware consumers can interpret the crate as a valid Workflow (Run) RO-Crate, while ISA-aware tools can traverse the same graph using laboratory-centric concepts. By unifying these perspectives, the proposed approach supports integrated traceability, richer metadata annotation, and cross-domain queries over complete research processes, advancing the practical realization of FDOs.

## 2 Related works

RO-Crate has been positioned as a general packaging model for FAIR research objects [2], 3]. WROC constrains RO-Crate for executable workflows and registry ingestion by WorkflowHub [4], 10], while WRROC captures retrospective execution provenance with CreateAction-based traces [5]. The ISA RO-Crate profile brings ISA’s laboratory provenance model into RO-Crate [7].

W3C PROV provides a general provenance core (Entity, Activity, Agent) and relations for retrospective provenance interchange [11], 12]. Workflow-centric extensions such as ProvONE and *Open Provenance Model for Workflows* (OPMW) introduce additional classes and relations to explicitly model prospective alongside retrospective provenance, typically as directed graphs of processes and data dependencies [13], 14].

CWLProv was designed to improve interoperability of workflow-run provenance by combining PROV serializations with a Research Object/BagIt-style folder structure for *Common Workflow Language* (CWL) enactments [15].

The *Annotated Research Context* (ARC) is a research data management framework for multi-modal research projects that supports the incremental creation of FDOs across experimental and computational domains [16], [17], [18], [19]. Its user-facing representation, the ARC Scaffold, provides a defined folder structure in which experimental provenance is modeled using tabular ISA files, while computational analyses are captured using workflow descriptions such as CWL to enable reuse of computational tools. ARC further provides a machine-actionable representation of its project state through ARC RO-Crate, which to date incorporates the ISA RO-Crate profile to represent the experimental dimension; the present work extends this RO-Crate representation with computational workflow and workflow-run provenance by aligning Workflow (Run) RO-Crate with the ISA-based process model.

## 3 Specification

The ARC WRROC profiles form a collection of specifications for describing both *prospective* provenance (i.e. workflow definitions yet to be executed, laboratory *Standard Operating Procedure*s (SOPs) used in the investigation) and *retrospective* provenance (i.e. completed runs of a workflow, performed experiment in a lab) across both experimental and computational domains. These profiles are modular by design: they can be reused in other profile collections and are not tied to the description of an RO-Crate’s root entity.

**Figure 1:**
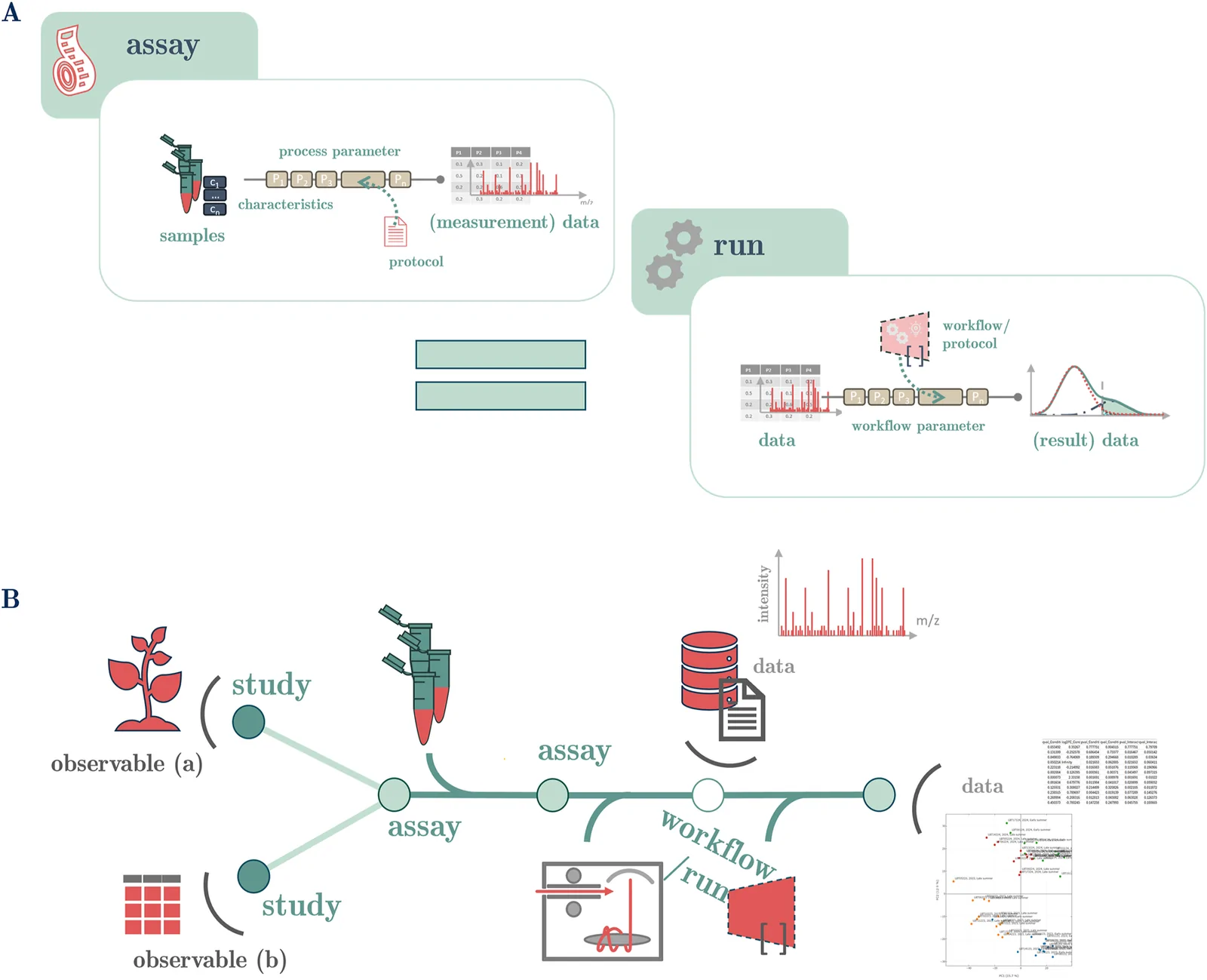
Unified provenance graph and workflow/assay similarity: (A) ISA tracks the provenance along the provenance graph. The metadata describing assays can be analogously applied to workflow runs. This enables the semantically rich and intuitive description from ISA to be applied to workflow executions. (B) The identity of a result lies largely in the metadata describing its provenance. It contains information about the origin and the process from there to the final output. The unification of the provenance graph facilitates reusability and queryability of the annotated data.

Computational and laboratory workflows share a common conceptual structure as seen in Figure 1, differing mainly in how they are carried out. Experimental provenance modeled using the ISA RO-Crate distinguishes between workflow descriptions (LabProtocol) and their actual executions (LabProcess). Both types support the annotation of parameterized metadata as key-value pairs, semantically determined and enriched through ontology terms. Computational workflows follow the same pattern: a workflow specification provides the protocol, and a workflow run corresponds to its execution. The ARC WRROC profiles extend established WROC and WRROC profiles so that they share the same process model as ISA, enabling seamless integration of computational and laboratory provenance. This unified model facilitates consistent provenance queries, metadata enrichment, and integrated visualization across domains.

To achieve this, the ARC WRROC profiles combine and extend existing RO-Crate profiles, in particular the WRROC profile collection. ARC WRROC enriches these with additional metadata fields and terminology aligned with the ISA RO-Crate profile. Its main purpose is to harmonize workflows and runs from the WRROC collection with the ISA concepts of LabProtocol and LabProcess, yielding a coherent process model that captures both prospective and retrospective provenance for computational and laboratory workflows. To facilitate incremental creation of FDOs, ARC WRROC relaxes some of the stricter requirements of its parent profiles. At the same time, we ensure that full compatibility with underlying profiles is maintained by composing mandatory underlying properties from mandatory and recommended properties in our profiles [20].

**Figure 2:**
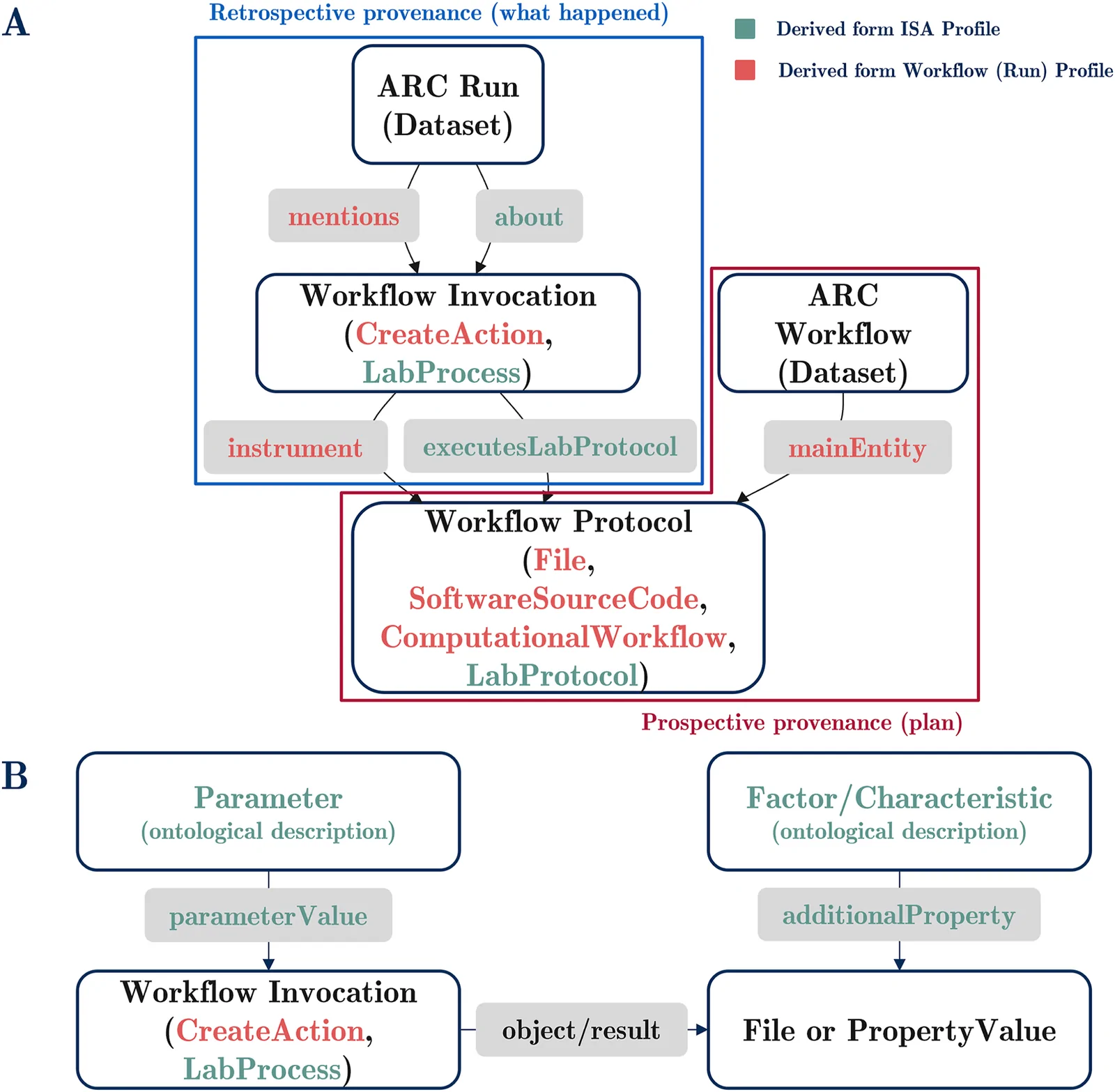
*Annotated Research Context Workflow Run RO-Crate* (ARC WRROC) entities and their relationship: (A) Workflow Protocol (prospective provenance) and Workflow Invocation (retrospective provenance) are the central entities aligned with their counterparts in the *Investigation Study Assay* (ISA) provenance graph via multi-typing with the *Workflow RO-Crate* (WROC)/*Workflow Run RO-Crate* (WRROC) equivalents. ARC Workflow and ARC Run are dataset container entities to enrich Workflow Protocols and Workflow Invocations with ISA-aligned metadata respectively. (B) Result objects may be further annotated via ISA Factor or Characteristic, while the create process (CreateAction/LabProcess) itself can be annotated using ISA Parameter.

The ARC WRROC specification defines four core entity profiles, whose relation is also visualized in Figure 2:

– **Workflow Protocol** – Encodes the prospective metadata of a workflow, including descriptions of inputs and outputs. It is a multi-typed entity (File, SoftwareSourceCode, ComputationalWorkflow, and LabProtocol), uniting computational and laboratory workflow descriptions. A Workflow Protocol can serve as the main entity of a WROC conforming to the Bioschemas ComputationalWorkflow profile, extended with ISA LabProtocol metadata.
– **ARC Workflow** – A Dataset container object representing a workflow folder inside an ARC. It can enrich a Workflow Protocol with additional ISA metadata, contains a mainEntity following the Workflow Protocol profile that describes the main workflow of the folder (analogous to WROCs), and may itself constitute a valid WROC (see Section 3.5 – Profile Compatibility).
– **Workflow Invocation** – A multi-typed entity (CreateAction and LabProcess) representing the execution of a Workflow Protocol. It records the invocation of a workflow with concrete values assigned to the protocol’s input parameters, thereby linking prospective definitions to retrospective provenance.
– **ARC Run** – A Dataset container object representing a run folder inside an ARC. It provides retrospective provenance, can enrich Workflow Invocations with additional ISA metadata, and may include files produced or consumed during the run.

### 3.1 Workflow Protocol

**Figure 3:**
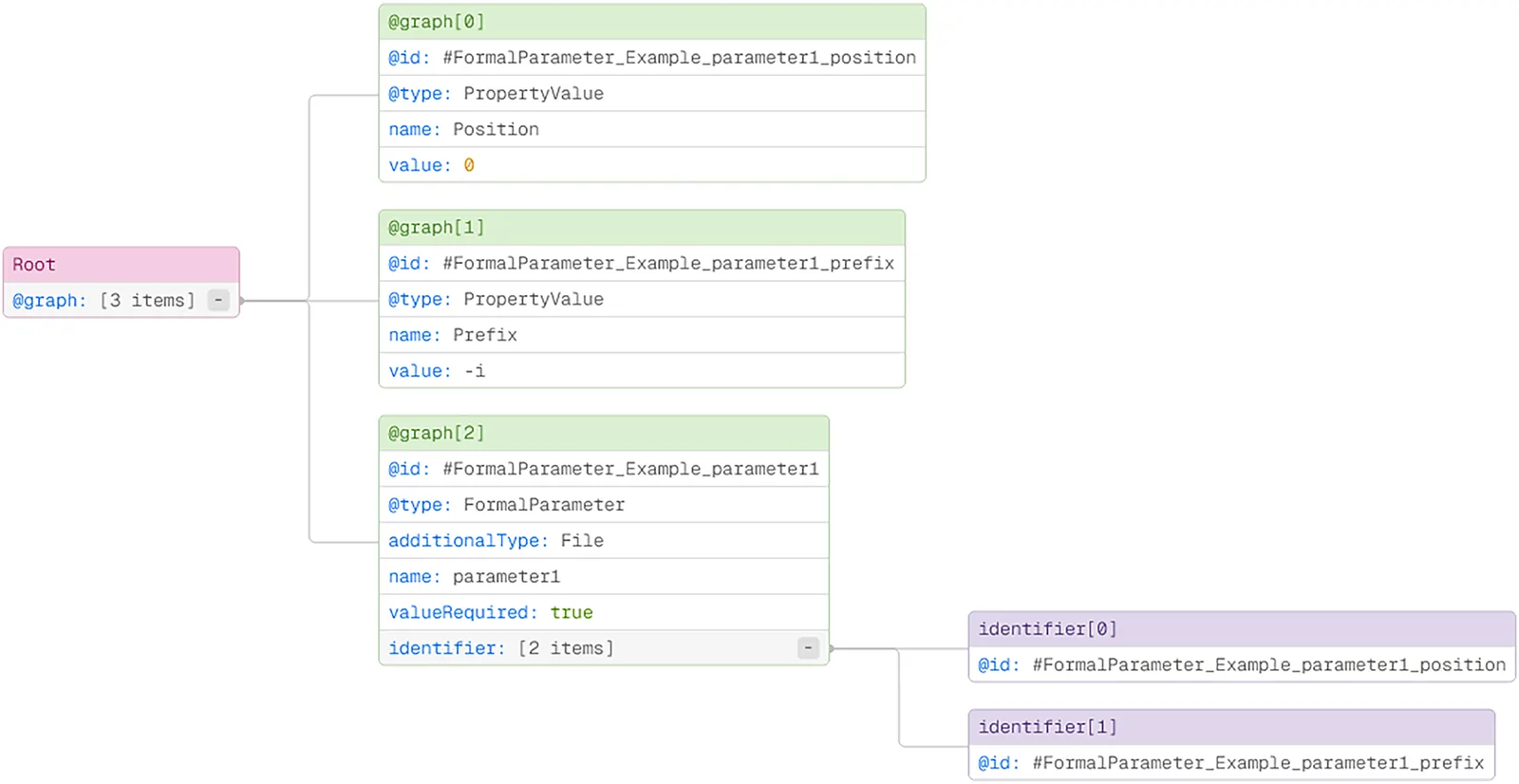
Parameter position and prefix encoding: the position and prefix of a parameter can be annotated through PropertyValues which are referenced in the identifier of the input parameter.

A multi-type object that combines ComputationalWorkflow and LabProtocol, providing prospective provenance of computational workflows that can be understood as traditional workflows as well as from a protocol (SOP) perspective. To encode CLI semantics for inputs, each associated FormalParameter identifier *MUST* be a PropertyValue whose name is either Prefix or Position, and whose value is a string (e.g., ‘−i’) or integer (e.g., ‘1’) describing prefix or position. Prefix and position may be included or omitted as appropriate for the invoked task. This enables automatic invocation from the Workflow Protocol. A resulting example JSON-LD tree view snippet can be seen in Figure 3.

### 3.2 ARC Workflow

An ARC Workflow is an object that bundles ISA-compliant administrative metadata (e.g., the person that created it) and the prospective provenance of the workflow in form of Workflow Protocol(s). It is based upon schema.org/Dataset and maps to the ISA-XLSX Workflow. In the context of an ARC, an ARC Workflow can be seen as the top-level object describing the contents and provenance of a single folder inside the workflow folder.

An ARC Workflow contains a mainEntity following the Workflow Protocol profile that describes the Main Workflow of this workflow folder, analogous to *Workflow RO-Crate*s (WROCs).

### 3.3 Workflow Invocation

A multi-type object that combines CreateAction and LabProcess, providing retrospective provenance of ComputationalWorkflow execution that can be understood as traditional workflow runs as well as from a process sequence perspective. The Workflow Invocation provides a mapping from concrete input to concrete output entities, defined through the object and result properties, respectively.

Workflow Invocations can be semantically enriched through Factors, which describe independent variables that determine the specific output of the experiment when processes and analysis were identical, Characteristics, which describe inherent properties of the source material, and Parameters, which describe steps in the workflow. Factors are attached to the outputs and Characteristics to the inputs while parameters are attached directly to the Workflow Invocation.

### 3.4 ARC Run

**Figure 4:**
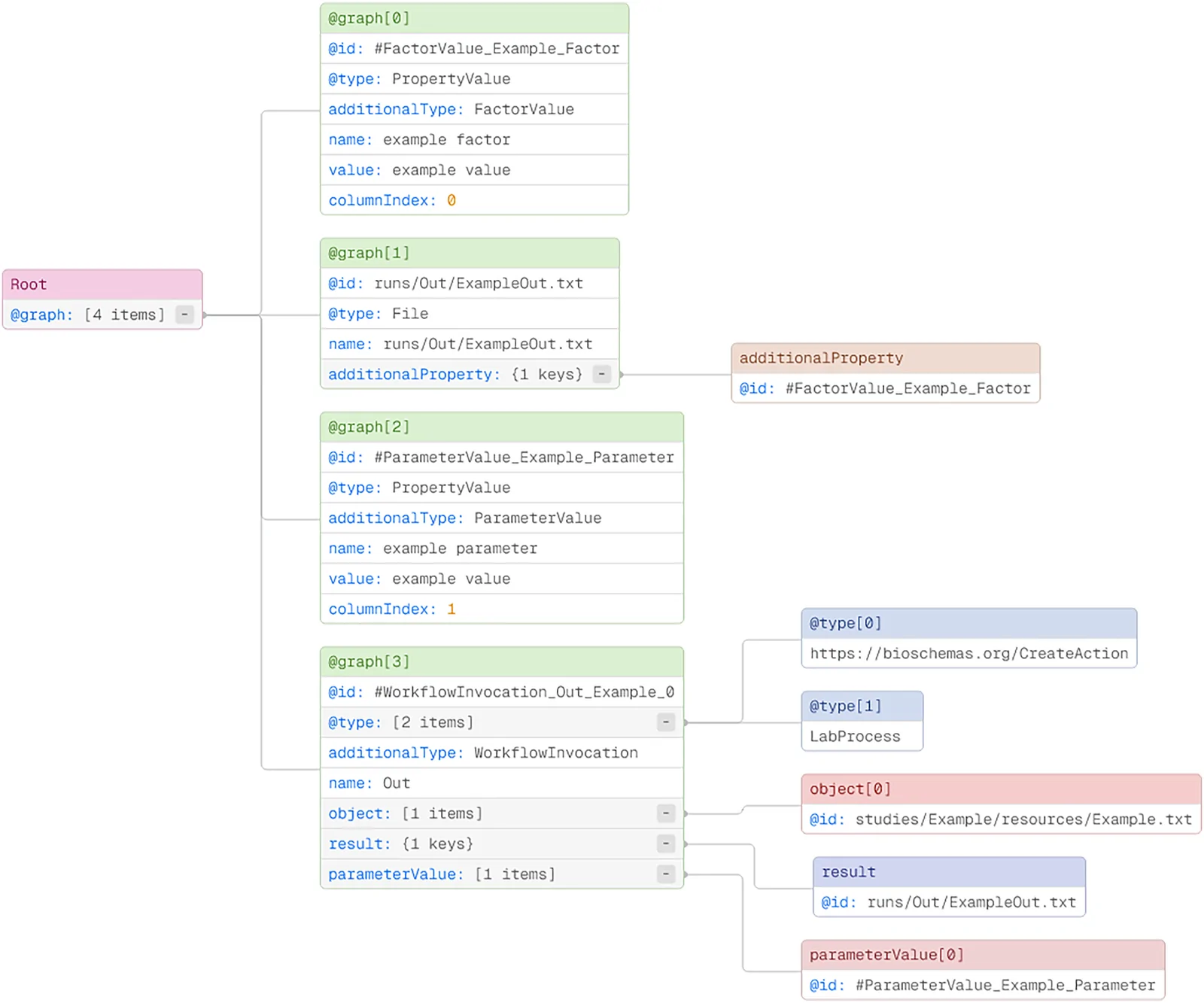
Provenance tracking via LabProcess multi-typing: result objects may be annotated via PropertyValue. Their additionalType is constrained to ISA Factor, Property, or Characteristic [7]. Factors and Characteristics attach to nodes (e.g., output files) via additionalProperty, while Parameters attach to edges (the LabProcess/CreateAction multi-type).

An ARC Run is an object that bundles ISA-compliant administrative metadata (e.g., the person that executed it) and the retrospective provenance of the run in form of Workflow Invocation(s). It is based upon schema.org/Dataset and maps to the ISA-XLSX Run. Figure 4 visualizes an example JSON-LD snippet showing the connections of the annotations to the nodes and edges. The multi-typing on the Workflow Invocation entities enables workflow executions to be annotated following the same principles as the LabProtocol executions in ISA.

### 3.5 Profile Compatibility

One aspect of ARCs is the support for gradually improving FAIR data annotation. The degree of FAIRness is not fixed but can evolve over time, reflecting a compromise between agile data development and the long-term stability of scholarly outputs. Therefore, the ARC WRROC has a minimal set of requirements, containing only the absolutely necessary properties [20]. This reduces the entry barrier in the creation of an ARC, but also encourages working towards a more detailed annotation. The next level of adoption is providing all recommended properties in this profile, which in turn contains all properties required in a Workflow (Run) RO-Crate [4], 5]. The ARC WRROC is then also a valid Workflow (Run) RO-Crate that utilizes the already existing community and tool support for this profile. This also works in the other direction, which then in turn allows those crates to use the existing ARC infrastructure and publication pipeline with only slight additions, as well as fine-grained annotation through ISA. The example run execution of the ProteomIQon tool chain and its corresponding WRROC and WROC with ro-crate-metadata.json is a proof-of-concept for this bi-directionality, as it contains the exported run and workflow parts of an ARC submitted to WorkflowHub [10], [21], [22], [23], [24]. The ro-crate-metadata.json contains all data relevant for WorkflowHub, as well as the ISA annotations. The ISA annotations are not yet acted upon by WorkflowHub, but can be shared there and re-imported into the ARC ecosystem.

## 4 Application

The primary application of ARC WRROC is the production of human-understandable retrospective annotations for computational analyses, including lightweight scripting and ad-hoc tool usage. It enables researchers to document and annotate analyses or simulations automatically or manually in the form of structured workflow runs. This produces enriched metadata that not only captures the technical details of a computation but also facilitates searchability and filtering across objectives, parameters, and intuitively described properties spanning projects and platforms.

A key advantage of this approach is that the annotation mechanism mirrors the conventions already familiar to experimental scientists from wet-lab documentation. Parameters, factors, and characteristics can be expressed in the same table-style representation as assays, lowering the barrier for researchers to create expressive workflow annotations. As a result, experimentalists can both read and write workflow descriptions using concepts they already know, effectively bridging the gap between computational and experimental provenance.

The alignment with the ISA standard further enables type-safe queries that seamlessly span wet-lab and dry-lab domains. Researchers can explore the full provenance cascade – from biological source material through computational processing – without requiring additional knowledge of the internal structure of the FDO.

ARC WRROC directly integrates into the mature ARC research data management tooling ecosystem. The primary ARC management software library ARCtrl [19] contains a reference implementation for creating and consuming RO-Crates following the ARC WRROC profile, enabling support in various downstream tools: ARCs submitted to DataHUB instances (cloud-based cooperative ARC management platforms) have ARC WRROCs attached to each commit [16]; Data publications to the ARChive (an Invenio-based publication platform) are defined by the metadata in these crates [24]; ARCitect (an ARC management graphical user interface application) supports manual annotation within the same tooling environment [17]; ARC validation and quality control services ensure domain-specific syntactic and semantic correctness during the research process.

Finally, ARC WRROC’s workflow packaging aligns with WROC requirements, most notably by declaring the main ComputationalWorkflow and its programmingLanguage. This ensures that crates are ingestible by WorkflowHub and can benefit from its registry ecosystem [4], 10]. The profile is also compatible with Galaxy, which already consumes workflow descriptions from WorkflowHub and exports workflow executions as WRROC. Designed with Galaxy’s execution methods in mind, the profile supports both import and export, including associated datasets, enabling Galaxy to directly produce workflow-based ARCs [25].

## 5 Discussion

### 5.1 Summary of contributions

The ARC WRROC profile introduced in this work aligns the ISA RO-Crate approach to laboratory processes and the WROC and WRROC representations for computational workflows and their executions. This results in a single, queryable metadata graph across experimental and computational spheres while retaining workflow-specific properties [20]. In doing so, ARC WRROC remains compatible with WorkflowHub’s WROC and the WRROC expectations, while preserving the ARC ecosystem’s emphasis on minimal viable annotation with a pathway to richer compatibility as needed [4], 5], 20].

### 5.2 Integration of prospective and retrospective provenance via multi-typing

A core contribution is the unification of prospective and retrospective provenance within one RO-Crate, achieved via multi-typing of entities to reflect both sides of the protocol-process divide. In WRROC, retrospective computational execution is centered on CreateAction, which links an execution to its describing workflow, inputs, and outputs [5]. ISA RO-Crate, conversely, uses LabProtocol to capture planned activities and LabProcess to record the actual laboratory processes connecting inputs and outputs, as well as their derivations [7]. Similarly, a workflow file can be described as both ComputationalWorkflow and LabProtocol to signal its dual role as an executable specification and a procedural description, satisfying WROC’s requirement that the crate’s mainEntity be a ComputationalWorkflow while enabling downstream experimental alignment [4], 20].

This multi-typing strategy leverages the RO-Crate profile’s mechanism – “a set of conventions, types and properties that one minimally can require and expect to be present” – to support reliable consumption by tools across domains.

### 5.3 Interoperability and alignment with RO-Crate and ISA (and WorkflowHub/WRROC)

ARC-WR RO-Crate’s interoperability derives from strict reuse of existing profile constraints where possible. RO-Crate provides the packaging model (a Dataset rooted crate with a ro-crate-metadata.json in JSON-LD) and encourages domain-specific profiles for machine consumption; ARC WRROC is positioned as such a profile, inheriting RO-Crate’s reliance on schema.org and JSON-LD for linked, portable metadata [3]. From WROC, ARC WRROC derives the requirement that the main workflow is a ComputationalWorkflow referenced by mainEntity and that recognized workflow languages (e.g., CWL, Galaxy, Nextflow) are declared via programmingLanguage, satisfying WorkflowHub’s ingestion and display expectations [4]. From WRROC, ARC WRROC adopts the principle that runs are expressed as CreateAction executions of the main workflow (instrument), thereby capturing retrospective computational provenance consistent with WRROC’s normative statements that a WRROC *MUST* have a ComputationalWorkflow main entity and *MUST* describe corresponding CreateAction instances [5].

From ISA RO-Crate, ARC WRROC derives the use of LabProtocol/LabProcess to describe assay protocols and performed processes [7]. This enables ARC WRROCs to co-locate computational workflows and assay descriptions, facilitating conversion between ARC and RO-Crate ecosystems. The resulting design allows a WROC – compatible submission to WorkflowHub while maintaining ARC’s broader experimental context, thus addressing both registry expectations and laboratory data management needs while also providing a unified provenance graph enabling querying not only one domain at a time, but both at once. Parameters relevant to laboratory protocols can be represented alongside computational parameters using the same LabProtocol/LabProcess and CreateAction graph, improving cross-domain queryability (e.g., “find all runs derived from samples in assay *X* that used workflow *Y* with parameter *Z*”) [4], 20].

### 5.4 Implications for FAIR and FDO practices (traceability, automation, reuse)

ARC-WR RO-Crate’s unified graph advances FAIRness by improving findability (search in both domains simultaneously and interlinked), interoperability (JSON-LD with community profiles), and reusability (linked provenance connecting samples, data, workflows, runs, and parameters). The FAIR Principles emphasize machine-actionability as a first-class requirement; by constraining types/properties through profiles and using established profiles for workflows and parameters, ARC WRROC increases the reliability of automated discovery and reuse [1]. Moreover, RO-Crate has articulated its compatibility with FAIR Digital Object practices, enabling ARC WRROCs to act as machine-actionable FDOs that expose both prospective and retrospective facets of a research object [3].

In particular, the WRROC stack demonstrates that crate metadata can capture both prospective (workflow description) and retrospective (execution provenance) aspects in one JSON-LD file, a practice that ARC WRROC extends across the experimental – computational boundary by multi-typing and profile alignment [5], 20]. For reuse, this permits consumers to reconstruct (or re-execute) computational analysis steps in context of the original assay protocols and parameterization, supporting repeatable and auditable Research Data Management workflows.

### 5.5 Comparison with related work and tools

#### 5.5.1 Workflow RO-Crate and WorkflowHub

Workflow RO-Crate and WorkflowHub focus on packaging a single ComputationalWorkflow (plus ancillary files) for exchange and registry ingestion; the profile mandates a mainEntity ComputationalWorkflow and recognized programmingLanguage instances (e.g., CWL, Galaxy, Nextflow), and constrains serialization to .crate.zip for interoperability [4]. WRROC extends this by requiring that executions be modeled as CreateAction linked via instrument to the main ComputationalWorkflow, capturing retrospective provenance of runs and (in more detailed variants) of internal steps [5]. ISA RO-Crate, in turn, provides an ISA-framed view of investigations, studies, assays, and their laboratory protocols/processes; its profile descriptions emphasize LabProcess as the core entity for derivations and parameters [7].

ARC-WR RO-Crate’s novelty lies in simultaneously meeting these expectations: it remains compatible to platforms such as WorkflowHub as a valid WROC (through mainEntity and supported languages), while also being consumable by ISA-aware tools through LabProtocol/LabProcess annotations and ISA-organized datasets [10]; Compared with approaches that keep laboratory and computational crates separate, ARC WRROC’s multi-typed, single-graph strategy reduces the cognitive and engineering overhead of linking across crates and avoids divergence between protocol definitions and executable workflows. It also enables uniform, type-consistent queries over the complete provenance graph and supports consumption by domain-specific experimental and computational tools as well as search engines.

#### 5.5.2 PROV-based workflow provenance and CWLProv

W3C PROV’s graph-centric ontology approach is powerful for formal reasoning over execution traces, but comes with two practical interoperability challenges: extensions are not directly interoperable due to differing granularities and assumptions, and *Workflow Management System* (WMS) support is often narrow (commonly one system per model) [5]. In other words, adopting a PROV extension usually implies committing to a particular modeling “dialect” and to the provenance output capabilities of specific workflow systems.

RO-Crate takes a different stance: it adopts a *packaging-first* representation (RO-Crate JSON-LD with schema.org) and uses profiles to constrain how prospective and retrospective provenance are expressed, rather than introducing a new provenance ontology. ARC WRROC’s multi-typing deliberately aligns computational workflow semantics with ISA’s protocol/process divide, enabling one coherent graph to serve both workflow-aware and ISA-aware consumers. Compared to PROV extensions, which primarily harmonize within the computational provenance space, ARC WRROC’s distinctive contribution is to make this alignment *structural* and queryable across domains (e.g., connecting assay-derived materials and factors with computational parameters and outputs) without requiring consumers to translate between separate experimental and computational provenance graphs [5], 7], 20].

Granularity further separates the approaches. PROV extensions commonly aim to model workflow structure and can represent executions at multiple abstraction levels, but the chosen extension typically prescribes what constitutes a “step” and how intermediate artifacts are represented [13], 14]. ARC WRROC, in contrast, makes granularity a *profile-guided* choice: at minimum, a WMS (or even ad-hoc scripting) can emit a single end-to-end CreateAction/LabProcess invocation with declared inputs/outputs, while richer traces can incrementally add recommended properties and additional actions to reach full Workflow (Run) RO-Crate compatibility. This reflects ARC’s “minimal requirements → recommended properties → full compatibility” pathway and reduces the requirement that every environment must expose fine-grained internal execution structure to participate [5], 20].

CWLProv explicitly includes both prospective and retrospective provenance, and in practice the reference implementation (cwltool) captures detailed step-level provenance with intermediate data and nested workflows (a high level of granularity) [15]. However, there are important limitations for interoperability and consumption: although CWLProv includes a plan within PROV, implementations often omit tool definitions and file formats, so consumers may need to inspect the native workflow definition to reconstruct full prospective provenance; CWLProv may include multiple metadata files and PROV serializations in different formats, complicating generation and consumption; and its high granularity requires substantial WMS support and has corresponded to low adoption (at the time of writing supported only by cwltool) [5].

ARC-WR RO-Crate addresses these issues by prioritizing *single-graph, profile-constrained packaging* over multi-file provenance bundles. Prospective and retrospective provenance are expressed directly in RO-Crate JSON-LD using established Workflow (Run) RO-Crate patterns (e.g., ComputationalWorkflow as mainEntity; runs as CreateAction linked via instrument) while simultaneously making the same entities usable as LabProtocol/LabProcess for ISA-aligned annotation and cross-domain traversal [4], 5], 7]. This also changes what is required from a WMS: ARC WRROC can accommodate systems that can only report coarse run-level provenance (or manual annotation), yet it remains interoperable with WorkflowHub expectations once recommended properties are provided, preserving exchangeability with existing WROC ecosystems [4], 20]. Finally, ARC WRROC’s modular profile design (not tied to the crate root entity) enables composing workflow/run provenance with ARC folder containers (ARC Workflow, ARC Run) and ISA-organized datasets, yielding an integrated experimental and computational provenance graph that supports cross-domain queries without forcing a consumer to reconcile separate “lab” and “workflow” bundles post hoc.

### 5.6 Versioning strategy

The ARC WRROC profiles follow *Semantic Versioning* (SemVer) to make changes predictable for both producers and consumers of crates. Each profile release is versioned independently and recorded in the profile metadata to support validation and long-term interpretability of archived crates.

Because ARC WRROC is designed to remain compatible with its parent profiles, its evolution is coupled to upstream developments in Workflow (Run) RO-Crate and ISA RO-Crate. We therefore track new releases of these specifications and update ARC WRROC accordingly, prioritizing compatibility-preserving updates where possible and issuing a major version only when upstream changes or accumulated community requirements necessitate breaking adjustments. Community feedback is incorporated transparently via GitHub issues and pull requests, which serve as the primary mechanism for reporting ambiguities, requesting extensions, and discussing interoperability with WMS and tooling.
